# Using Photogrammetry to Analyze Anatomical Changes in the Nipple in Juvenile and Adolescent Scoliosis Patients

**DOI:** 10.1007/s00266-024-04039-5

**Published:** 2024-04-30

**Authors:** Ozden Bedre Duygu, Figen Govsa, Ahmet Bicer

**Affiliations:** 1 Department of Anatomy, Digital Imaging and 3D Modelling Laboratory, Faculty of Medicine, Ege University, 35100 Izmir, Turkey; 2 Department of Anatomy, Faculty of Medicine, Izmir Bakircay University, Izmir, Turkey; 3 Department Plastic and Reconstructive Surgery, Faculty of Medicine, Ege University, Izmir, Turkey

**Keywords:** Breast asymmetry, Surface topography, Anterior trunk asymmetry, Breast surface anatomy, Photogrammetric analysis, Torsional deformity

## Abstract

**Background:**

The need for an objective set of anterior trunk measurements, such as nipple and clavicular shoulder joints, is essential to quantify the anterior asymmetry present in scoliosis. This study aims to characterize breast asymmetry (BA) in young individuals with scoliosis using photogrammetry.

**Methods:**

Digital photographs of the anterior trunk of the 51 scoliosis patients aged 4–20 years were taken from an anterior perspective. These images were then transferred to a computer. Ten parameters were measured using the ImageJ software. The positions of patients’ nipples were classified into 6 types based on ratios on the *x*-axis.

**Results:**

The majority of patients had a right breast that was larger, intensifying the apparent BA due to trunk rotation. The apical vertebra level in patients was found at T8 in 23.6% and T9 in 45.1%. In 92.5% of the patients, the right breast was consistently larger. The lengths between the lateral boundaries and nipples of the right and left breasts and between the medial boundary and nipple of the right breast were statistically significantly higher in males than in females (*p* < 0.05). Significant differences were found when comparing the values of the lengths between the medial boundaries and nipples of the right and left breasts, the difference in length between the right and left acromioclavicular joint lines, and the angles of the nipple and acromioclavicular joint with the degrees of scoliosis in juvenile and adolescents (*p* < 0.05). Pearson regression analysis revealed a significant correlation between BA differences and the Cobb angle with a correlation coefficient of 0.901. Factors related to breast aesthetics, like differences in the height of nipples and the distance from the sternal notch to the nipple, represent 30% of the overall score.

**Conclusion:**

The study concluded that there is a significant correlation between the severity of scoliosis and BA differences. Augmentation mammaplasty for BA not only decreased the breast difference but also leveled the nipple disparities. Photogrammetry is considered to be an alternative to other methods and is believed to contribute to the follow-up of BA.

**Level of Evidence IV:**

This journal requires that authors assign a level of evidence to each article. For a full description of these Evidence-Based Medicine ratings, please refer to the Table of Contents or the online Instructions to Authors www.springer.com/00266.

## Introduction

Adolescent idiopathic scoliosis (AIS) represents a multifaceted three-dimensional torsional deformity of the spine, frequently accompanied by unilateral rib protrusion, particularly evident in thoracic involvement [1–3]. This thoracic asymmetry observed in AIS is closely linked to breast asymmetry (BA), especially in Cobb angles exceeding 10° [4, 5]. The ideal trunk symmetry can be profoundly disrupted during the puberty phase, leading to an array of cosmetic concerns [6, 7]. AIS has the potential to induce considerable disability, profoundly affecting a person's self-perception, self-worth, and social interactions [1, 8–10].

Denoel et al.'s anthropometric investigation consistently illustrated a heightened prevalence of BA in adolescent girls with AIS [11]. The convex side breast was predominantly characterized by a reduced volume, a shorter and more cranially positioned mammary base, a diminished sternomammary distance, and a smaller areola [4, 12]. However, despite the extensive literature on this subject, current insights regarding the relationship between the magnitude of scoliosis curve and the severity of BA remain inconsistent, with no unified consensus in sight. This ambiguity is largely attributed to the lack of a standardized definition of BA and the diverse methodologies employed in different studies for breast measurements [13, 14].

Numerous studies have endeavored to objectively document the existence of BA and chest wall asymmetry in individuals with AIS [15–18]. The anterior chest wall displays considerable variability among AIS patients, even with comparable Cobb angles.

In recent years, researchers have increasingly focused on the anterior costal arch prominence's asymmetry [19–23]. The nipple, as a primary clinical indicator, appears to play a pivotal role in BA associated with AIS. Irrespective of the surgical approach, be it reconstructive or aesthetic, the primary objective remains the restoration of the breast's natural contour and alignment with patient aspirations. The congruence between one's physical aesthetics and their psychological body image critically influences the overall quality of life [24–27].

While the initial diagnosis of AIS is generally made incidentally by radiographs obtained for various reasons, a follow-up with X-rays bearing an increased cancer risk drove clinicians toward other means such as MRI, which is expensive and not immediately accessible [2, 18, 28, 29]. Photogrammetric surface topography methods, which enable swift and accurate breast imaging without radiation exposure or alteration to the body's surface, present a viable alternative in clinical environments [1]. Historically, surface topography has been employed to measure dorsal deformities in scoliosis, serving as a non-radiative evaluative instrument. However, less research has been conducted for quantifying the anterior deformities and their implications on body image, and quality of life of patients with AIS [30].

The aim of this study is to assess BA in younger AIS people and to elucidate their correlation with deformities of the anterior chest wall and the spine. This research also explored the relationship between the parameters of the breasts and the severity of the scoliosis.

## Materials and Methods

### Study Design

This descriptive morphometric study received approval from the Department of Anatomy, Faculty of Medicine, Ege University, and was conducted in the Digital Imaging and Three-dimensional Modelling Laboratory. The study encompassed 51 non-operated scoliotic young people, aged between 4 and 20 years, with an average follow-up period of 2 years. All participants were young and nulliparous, consisting of 25 girls and 26 boys. Ethical approval for the research was granted by the Human Research Ethics Committee affiliated with the authors' institutions, and informed consent was obtained from all participants (18–6.1/32).

**Fig. 1:**
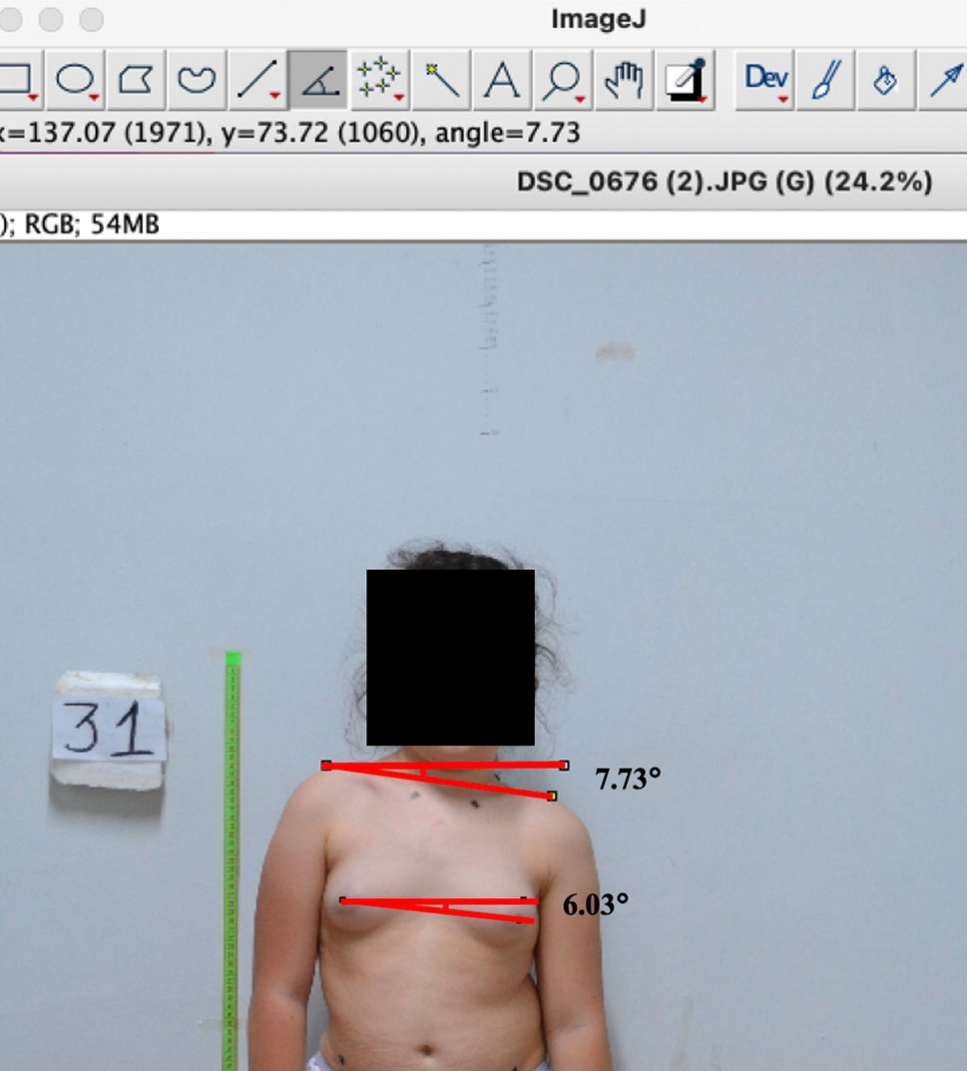
Photo-anthropometric measurement of the acromioclavicular joint angle (7.73°) and nipple angle (6.63°) in a scoliosis child with a 40° thoracic curvature

**Fig. 2:**
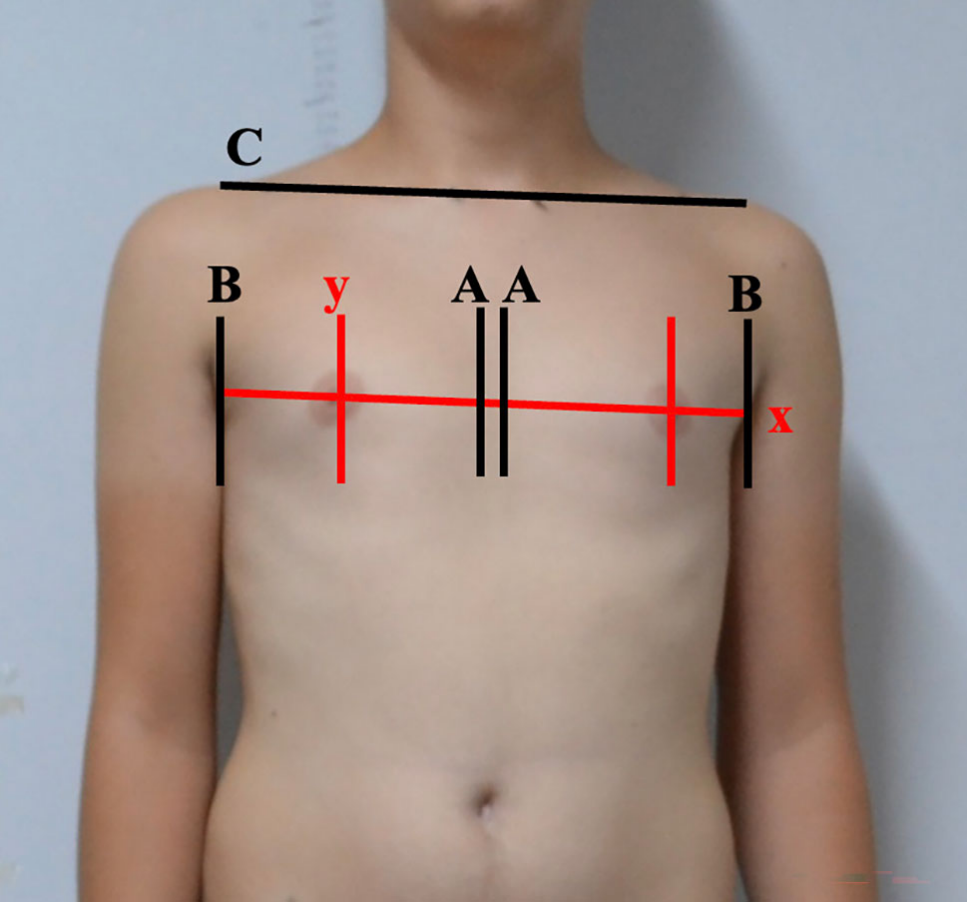
Depiction of anatomically marked points on the body and standard positions for the photo-anthropometric method. Lines defined for measurements. X: Horizontal line tangent to the nipple, Y: vertical line tangent to the nipple, **A** line passing through the medial boundary of the breast, **B** line passing through the lateral boundary of the breast, **C** line intersecting both right and left acromioclavicular joints

**Fig. 3:**
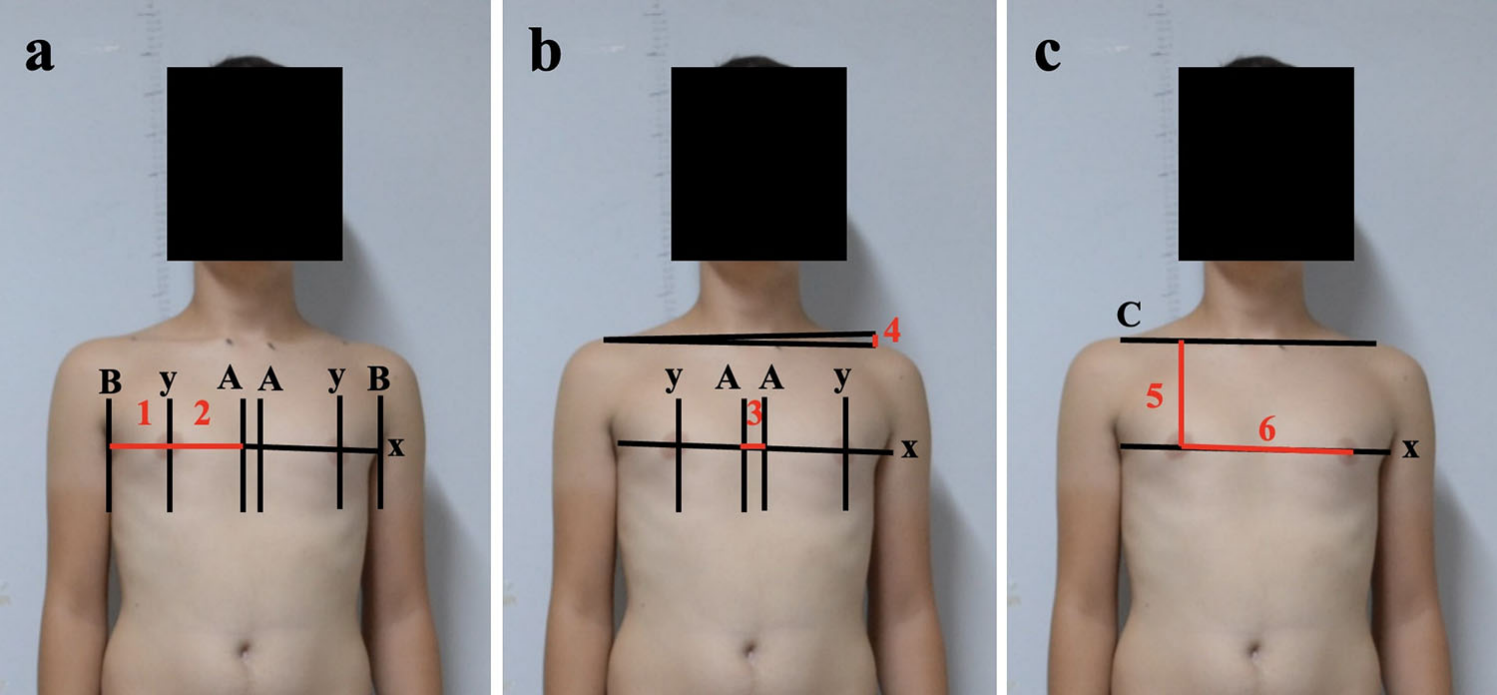
Length measurements. **1**: BY length measurement is the horizontal distance between the line drawn from the lateral boundary of the breast and the vertical line intersecting the nipple. **2**: AY length measurement is the horizontal distance between the line drawn from the medial boundary of the breast and the vertical line intersecting the nipple. **3**: AA length is the horizontal distance between the lines intersecting the medial boundaries of the right and left breasts. **4**: ACUF is the length between the line intersecting both right and left art. acromioclavicularis and the horizontal line.** 5**: MACU is the vertical distance between the nipple and the axis of the acromioclavicular joints. **6**: YYU is the distance between the right and left nipples

Participants were selected randomly from the outpatient population using a comprehensive randomization method. The enrollment criteria were as follows: (1) AIS people aged 4–20 years; (2) presenting with structural thoracic curves; (3) at C4; (4) with photographs taken in the anatomical position: standing upright with arms at their sides (Figs. 1, 2 and 3).

Exclusion criteria included: (1) patients who had undergone any surgical interventions leading to secondary or iatrogenic BA, such as tumor excision, augmentation or reduction mammaplasty, or cardiac and chest surgeries, as well as patients post-scoliosis treatment; (2) adolescents with anterior chest wall deformities, like pectus excavatum or pectus carinatum.

The degrees of thoracic curvature were determined using the Cobb method, applied to anteroposterior radiographic images (Tables 2 and 3).

### Duration of Research

The research duration included 1 month to determine the reference anatomical points and lines on the body, 6 months for data collection, 4 months for data evaluation using the ImageJ program, and 1 month for recording the data in the evaluation of anatomical changes in the nipple through the photogrammetric method in juvenile and adolescent idiopathic scoliosis.

### Study Environment Preparation Steps

A Nikon D3100 digital camera, a non-toxic colored marker, and a measuring tape were essential tools for this study. The measuring tape was placed vertically beside the adolescents. The camera was positioned 280 cm away from the patient on a tripod, with the camera height adjusted to 130 cm.

### Patient Positioning for Postural Evaluation

**Fig. 4:**
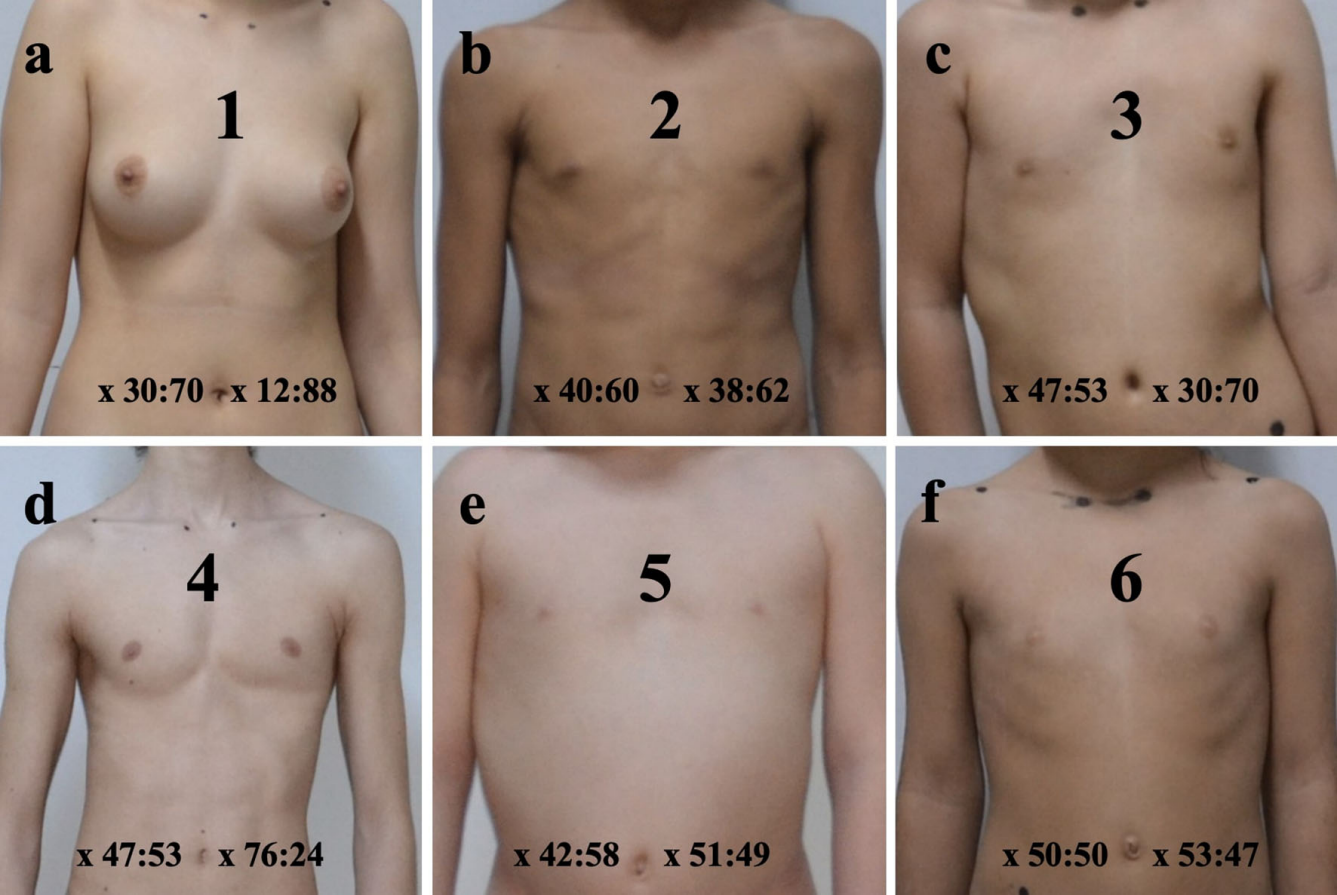
Six frontal images related to ratios on the *X*-axis (*x* = BY:YA). The scoliosis adolescents participating in our study were classified into 6 types based on ratios in the *X*-axis

A digital camera was used to take images. Key body reference points, such as the sternal facet of the clavicle and acromial facet of the clavicle, were marked using a non-toxic colored marker (Figs. 1, 2, 3 and 4).

### Data Collection Method of the Research

Digital photogrammetric images of the patients were transferred to an Apple MacBook Pro computer by a researcher from the Department of Anatomy. The distances and angles between the anatomical points identified in the study were measured using a standard method with the ImageJ software program.

### Using the ImageJ software program

Photogrammetric images of juvenile and adolescent idiopathic scoliosis patients were imported into the ImageJ program by selecting the "Open" option from the “File” menu. In the ImageJ program, a 10-cm line was drawn on the guide ruler based on the scale in each patient's image, and the pixel value of this length was recorded according to the program. Afterward, the guide ruler page was closed. The "Set Scale" option was accessed from the "Analyze" menu, and the pixel value was recorded. Subsequently, the researcher measured angles and lengths. These steps were performed individually for 51 scoliosis patients. The obtained length and angle measurements were recorded in the Excel program.

### Assessment of Photo-anthropometric Technique Parameters

Digital photographs of the anterior trunk of scoliosis patients were taken from an anterior perspective. The anterior profile view of both the concave and convex breasts was analyzed to assess various linear and angular morphological breast parameters described below (Figs. 1, 2 and 3). ImageJ software facilitated these measurements. A total of ten morphological parameters were utilized to estimate breast profile and symmetry by identifying several anthropological points.

These measurements included:

*AA Length Measurement* This measures the horizontal distance between the lines intersecting the medial boundaries of the right and left breasts (Figs. 2 and 3).

*AY Length Measurement* This signifies the horizontal distance between the line drawn from the medial boundary of the breast and the vertical line intersecting the nipple (Figs. 2 and 3).

*BY Length Measurement* This represents the horizontal distance between the line drawn from the lateral boundary of the breast and the vertical line intersecting the nipple.

*Acromioclavicular Uniformity Factor (ACUF)* This refers to the length between the lines intersecting both the right and left acromioclavicular joints and the horizontal axis (Figs. 1, 2 and 3).

*MACU Distance* This is the vertical space between the nipple and the line of the acromioclavicular joints (Figs. 2 and 3).

*YY Distance* The length between the right and left nipples (Figs. 1, 2 and 3).

*Nipple Angle* This angle is defined by the intersection of the line connecting both right and left nipples and the horizontal line.

*Acromioclavicular Joint Angle* The angle created between the acromioclavicular joint line and the horizontal axis.

The positioning of patients' nipples was classified into six types based on ratios (BY:AY) along the *x*-axis (Fig. 4). The breakdown of the right and left ratio is as follows: Type 1 (14%), Type 2 (39%), Type 3 (33%), Type 4 (4%), Type 5 (8%), and Type 6 (2%).

In this two-dimensional morphological analysis, using the sternal notch as a fixed anthropometric landmark and the nipples as two distinct mobile anthropometric points, we defined aggravated morphological BA.

### Statistical analysis

Data were statistically processed using SPSS software version 17.0 (SPSS, Inc, USA). Descriptive statistics were employed to evaluate patient demographics. Quantitative variables were represented as mean ± standard deviation (SD). The paired-sample *t*-test was utilized to assess the surgical alterations related to breast asymmetry. Additionally, the independent-sample *t*-test was implemented to discern differences between groups with exacerbated and mitigated BA. A *p* value less than 0.05 was considered statistically significant.

## Result

### Patient Demographics

The study included a total of 51 AIS patients, consisting of 26 boys and 25 girls, leading to a male-to-female ratio of 1:1. Of these patients, 11 had juvenile idiopathic scoliosis, and 40 had AIS. The average age of female participants was 10.56, while for males, it was 13.8 years. The average thoracic scoliosis degree was found to be 19.72° in females and 15.96° in males. The right breast length (11.94 ± 2.41 cm) was determined to be greater than the left, which measured 10.75 ± 2.07 cm. The thoracic curvature of the patients was oriented to the right. The rate of patients with their apical vertebra level at T6 was 11.7%, at T7 was 19.6%, at T8 was 23.6%, and those at T9 was 45.1%.

### Photo-anthropometric Measurements

The accuracy of photographic measurements in juvenile and adolescents with presentations of the anterior surface of the trunk is detailed in Table 1 and 2. The distance between the lateral boundary and the nipple of both right and left breasts, and the distance from the medial boundary to the nipple of the right breast were statistically significantly greater in boys compared to females (*p* < 0.05) (Table 1). When comparing the difference in distance between the medial boundaries of the right and left breasts and the nipple, the difference in distance between the right and left acromioclavicular lines, the nipple angle, and the acromioclavicular joint angle based on patients' scoliosis degrees, a statistically significant difference was observed (*p* < 0.05) (Figs. 1, 2 and 3) (Table 2). A moderate positive correlation was detected between the scoliosis degrees of the patients and the differences in distances between the right and left acromioclavicular lines, the nipple angle, and the acromioclavicular joint angle values (Figs. 1, 2 and 3) (Tables 2 and 3). Pearson correlation analysis revealed a significant association between the disparity in breast measurements on the two sides and the severity of scoliosis.

**Table 1:** Measurements of photo-anthropometric parameters segmented by gender in patients with scoliosis

Measurements/variables	Side	Girls	Boys	*p* value
*BY length*: The horizontal distance between the line drawn from the lateral boundary of the breast and the vertical line intersecting the nipple	Right	4.39 ± 1.06 cm	5.29 ± 1.2 cm	0.007*
	Left	3.39 ± 1.08 cm	4.43 ± 1.09 cm	0.001*
Difference in length between the right and left BY length		1 ± 1.27 cm	0.85 ± 1.09 cm	0.66
*AA length*: The horizontal distance between the lines intersecting the medial boundaries of the right and left breasts		3.73 ± 0.94 cm	4.27 ± 1.47 cm	0.12
*AY length*: The horizontal distance between the line drawn from the medial boundary of the breast and the vertical line intersecting the nipple	Right	6.56 ± 1.84 cm	7.6 ± 1.64 cm	0.03*
	Left	6.6 ± 1.87 cm	7.05 ± 1.77 cm	0.37
Difference in length between the right and left AY length		-0.03± 0.93 cm	0.54 ± 1.38 cm	0.08
Difference in length between the right and left acromioclavicular joint lines		1.21 ± 0.58 cm	1.03 ± 0.59 cm	0.29
The distance between the nipple and the line of the acromioclavicular joints	Right	13.32 ± 2.61 cm	14.15 ± 2.62 cm	0.26
	Left	12.97 ± 2.66 cm	14.13 ± 2.55 cm	0.11
*YY length*: The length between the right and left nipples		16.9 ± 3.95 cm	18.93 ± 3.63 cm	0.06
*Nipple angle*: the intersection of the line connecting both right and left nipples and the horizontal line		2.36° ± 1.56°	1.64° ± 0.95°	0.05*
*Acromioclavicular joint angle*: the angle created between the acromioclavicular joint line and the horizontal axis		2.51° ± 1.96°	1.66° ± 1.21°	0.08

Values are presented as mean and standard deviation. Paired t tests then were used to examine any differences between girls and boys mean values

*Results showed significant differences between girls and boys mean values (*p* < 0.05)

**Table 2:** Values of photo-anthropometric method parameters

Measurements/variables	Side	Cobbs degree		*p* value
		0°–20°	21°–40°	
*BY length*: The horizontal distance between the line drawn from the lateral boundary of the breast and the vertical line intersecting the nipple	Right	4.94 ± 1.18 cm	4.68 ± 1.28 cm	0.45
	Left	3.95 ± 1.18 cm	3.87 ± 1.25 cm	0.83
Difference in length between the right and left BY length		0.99 ± 1.08 cm	0.8 ± 1.34 cm	0.57
*AA length*: The horizontal distance between the lines intersecting the medial boundaries of the right and left breasts		3.88 ± 0.97 cm	4.21 ± 1.64 cm	0.36
*AY length*: The horizontal distance between the line drawn from the medial boundary of the breast and the vertical line intersecting the nipple	Right	6.93 ± 1.69 cm	7.37 ± 2 cm	0.4
	Left	6.98 ± 1.63 cm	6.57 ± 2.12 cm	0.43
Difference in length between the right and left AY length		− 0.05 ± 0.82 cm	0.8 ± 1.55 cm	0.01
Difference in length between the right and left acromioclavicular joint lines		0.94 ± 0.53 cm	1.43 ± 0.57 cm	0.003
The distance between the nipple and the line of the acromioclavicular joints	Right	13.82 ± 2.77 cm	13.61 ± 2.43 cm	0.78
	Left	13.73 ± 2.73 cm	13.27 ± 2.54 cm	0.55
*YY length*: the length between the right and left nipples		17.8 ± 3.66 cm	18.16 ± 4.35 cm	0.75
*Nipple angle*: The intersection of the line connecting both right and left nipples and the horizontal line		1.45° ± 0.76°	2.92° ± 1.56°	0.001
*Acromioclavicular joint angle*: The angle created between the acromioclavicular joint line and the horizontal axis		1.61° ± 1.15°	2.74° ± 1.93°	0.01

Values are presented as mean and standard deviation. Paired t tests then were used to examine any differences between girls and boys mean values

*Results showed significant differences between girls and boys mean values (*p* < 0.05)

**Table 3:** Pearson correlation analysis of the parameters

Measurements/variables	Side	Gender (*R*)	Age (*R*)	Cobbs degree (*R*)
*BY length*: The horizontal distance between the line drawn from the lateral boundary of the breast and the vertical line intersecting the nipple	Right	0.373	0.368	− 0.128
	Left	0.439	0.253	− 0.56
Difference in length between the right and left sides BY length		0.16	0.673	0.059
*AA length*: The horizontal distance between the lines intersecting the medial boundaries of the right and left breasts		0.215	0.532	0.257
*AY length*: The horizontal distance between the line drawn from the medial boundary of the breast and the vertical line intersecting the nipple	Right	0.29	0.672	0.132
	Left	0.126	0.513	− 0.044
Difference in length between the right and left AY length		0.243	0.232	0.263
Difference in length between the right and left acromioclavicular joints lines		− 0.149	0.264	0.529
The distance between the nipple and the line of the acromioclavicular joints	Right	0.160	0.673	0.059
	Left	0.222	0.734	− 0.012
*YY length*: The length between the right and left nipples		0.263	0.724	0.124
*Nipple angle*: The intersection of the line connecting both right and left nipples and the horizontal line		− 0.275	− 0.025	0.583
*Acromioclavicular joint angle*: The angle created between the acromioclavicular joint line and the horizontal axis		− 0.242	0.066	0.436

Values are presented as mean and standard deviation. Paired t tests then were used to examine any differences between girls and boys mean values

*Results showed significant differences between girls and boys mean values (*p* < 0.05)

Shoulder metrics, encompassing height disparity and shoulder slope variation, constitute 40% of the overall assessment. BA factors, such as variations in nipple elevation and the sternal notch-to-nipple distance, account for 30% of the overall score (Table 2). Waist asymmetry comprises the remaining 30, offering an objective and quantifiable evaluation of anterior trunk deformity

## Discussion

The intersection of juvenile and AIS and BA presents a nuanced facet of the scoliosis discourse, particularly emphasizing cosmetic implications and the resultant psychosocial impacts [3, 9]. Our study meticulously investigates this interplay, bringing to light the pervasive nature of BA among AIS patients and the subsequent inclination toward surgical interventions for aesthetic amelioration. Notably, the prevalence of breast size, volume, and positioning discrepancies was substantiated in a significant portion of AIS-affected females, aligning with prior research that underscored an elevated concern among adolescent males regarding atypical physical development and the potential for altered body image perceptions (Tables 1, 2 and 3, Figs. 1, 2, 3 and 4). This concern was paralleled by an increased anxiety toward peer relationships, spotlighting the profound influence of physical appearance on psychological well-being.

Knot et al. documented a 6.2° difference between the right and left shoulder slopes [31]. The acromion processes alignment is nearly horizontal at 0.1°. Collectively, these parameters account for 40% of the total asymmetry score in our study.

Amidst the backdrop of gender-specific concerns, our findings highlight an imperative consideration—the impact of scoliosis on the anterior trunk and, by extension, breast morphology. The thoracic cage's asymmetrical elliptical surface deformity, accentuated during puberty, lays the groundwork for the structural deformities observed [18, 20, 31–33]. This study has illustrated that the severity of scoliosis, quantified through Cobb's angle, correlates moderately with the degree of BA, evidenced by disparities in breast measurements on either side of the trunk (Figs. 1, 2, 3 and 4). Such asymmetries not only manifest in morphological changes but also significantly contribute to the psychosocial distress experienced by individuals, necessitating a deeper exploration into their interrelation (Figs. 1, 2, and 4a,c,f). Concerning the correlation between BA and scoliosis severity, findings range from 11.4 to 81.1% [14, 23]. Previous research has highlighted a myriad of asymmetrical alterations, pinpointing that the convex breast often has reduced volume, a diminished areola, a higher and shorter mammary base, and a decreased sternomammary distance [19, 20, 34–36]. BAs are identified manifest in various morphological modifications involving the breast, nipple–areola complex, or both in terms of shape, volume, and alignment [22, 27]. Specifically, BA variations comprise alterations in the nipple–areola complex (24%), volume (44%), base constriction (29%), inframammary fold positioning (30%), and grades 1–3 ptosis (29%).

Several studies have highlighted a mild positive correlation between BA and the Cobb angle in female AIS adolescents [14, 23, 34–39].

Atici and colleagues found that BA existed in 33.3% of patients with a thoracic scoliosis Cobb angle > 50°, while 66.7% of patients had a Cobb angle < 50° (*p* > 0.05) [37]. Additionally, Atici observed that the thoracic apical vertebra was identified as T7 in 25.0% of patients and T9 in 50% [37]. No statistically significant difference in BA was established between those with a thoracic apical vertebra of T9 and those with one positioned above T9 in this study (*p* > 0.05).

Given the cosmetic implications, several people have sought consultations for augmentation mammaplasty to achieve symmetric and aesthetically pleasing breasts [8, 9, 35]. Clinically, BA often goes unnoticed and is underestimated compared to the skeletal deformities prominently recognized by AIS adolescents. Many seeking augmentation mammaplasty are unaware of their BA, and almost all are oblivious to their scoliosis [14]. Initially, the most pronounced feature of scoliosis is the coronal spinal curvature; however, as the condition progresses, the sagittal alignment becomes more significant. Overlooking the anterior aspect of the rib cage can result in postoperative aesthetic complications, physiological distress, and exacerbation of BA [19]. Hence, there is an increasing emphasis on recognizing BA in AIS adolescents. Comprehensive evaluation, both posteriorly and anteriorly, is crucial. Photoanthropometry serves as an instrumental tool for measurement, with patients often exhibiting varying degrees of asymmetry due to uneven shoulders or postural issues (Tables 1, 2 and 3).

In AIS adolescents, those with the most rotated vertebrae, forming the skeletal foundation of both breasts [2, 4, 28], along with a compensatory BA pattern, are more susceptible to iatrogenic worsening of BA. It is widely understood that thoracic AIS juveniles with an apex above T7 are relatively rare [38].

In this study, both girls and boys were evaluated. The mean age was 10.56 for females and 13.8 for males. The average scoliosis angle was 19.72° for females and 15.96° for males. The calculated mean Cobb angle was 21.5°. A vast majority (92.5%) exhibited a more prominent right breast, amplifying the perceived BA due to trunk rotation. The apical vertebra was located at T8 in 23.6%, and T9 in 45.1%. In this population, the right breast was consistently larger. Measurements from anthropometric landmarks such as the acromioclavicular joint, breast and sternal notch, particularly the latter, revealed statistically significant differences (Tables 1, 2, 3). The distances between lateral boundaries and nipples of both breasts, and between the medial boundary and nipple of the right breast, were significantly greater in males (*p* < 0.05). Statistically significant disparities were observed when comparing various measurements related to BA and their association with scoliosis degrees (*p* < 0.05).

This phenomenon might be attributed to the combined effects of derotational coupling in the transverse plane and longitudinal extension in the coronal plane. Such effects counterbalance each other on the concave side, keeping the concave sternal notch–nipple tilt angle consistent. Regarding the surgical impact on the severity of morphological BA, both the concave and convex differences in clavicle–nipple length and sternal notch–nipple tilt angle remained significantly divergent from their pre-operative values (Fig. 3C-5). Factors related to breast aesthetics, like differences in the height of nipples and the distance from the sternal notch to the nipple, represent 30% of the overall score (Table 2). The final 30% is attributed to asymmetry in the waist, providing a measurable assessment of deformity in the front part of the trunk.

From a morphological perspective, anterior views of topless patient photographs, taken in an anatomical position preoperatively, were consistently used to assess the cosmetic outcomes of scoliosis surgery (Figs. 1, 2, 3 and 4). These images served as valuable resources for evaluating the morphological changes in BA post-surgery in this observational study.

Our collaborative research endeavor, engaging a multidisciplinary team comprising surface topography anatomists, scoliosis spine surgeons, and plastic surgeons, emphasizes the significance of comprehensive evaluation in AIS management. By incorporating photoanthropometry and surface topography analysis, we advocate for a more inclusive assessment strategy that captures the full spectrum of AIS-induced deformities, thereby facilitating targeted interventions.

Our study, despite its limitations, serves as a pivotal step toward understanding and addressing the complex dynamics between scoliosis severity and breast asymmetry, paving the way for more refined and patient-centric therapeutic modalities.

Furthermore, the surgical correction of AIS, while primarily aimed at rectifying spinal curvature, inadvertently accentuates BA, as indicated by our observational analysis of postoperative outcomes. This revelation underscores the critical need for a holistic approach in managing AIS, one that extends beyond mere spinal alignment to encompass the anterior trunk's cosmetic concerns. The surgical community, particularly those specializing in breast reconstruction and augmentation mammaplasty, must be cognizant of these nuances to address patient apprehensions effectively and improve overall quality of life.

## Conclusion

The intersection of AIS and BA is marked by significant clinical and psychosocial implications, warranting a paradigm shift in treatment approaches. The advent of surgical augmentation as a means to rectify BA highlights the critical role of cosmetic considerations in the broader AIS management spectrum. As we move forward, it is imperative that longitudinal studies be conducted to validate the efficacy of integrated surgical and medical strategies, ensuring the sustained well-being and satisfaction of AIS patients.
